# Influence of Implant Impression Methods, Polymer Materials, and Implant Angulation on the Accuracy of Dental Models

**DOI:** 10.3390/polym14142821

**Published:** 2022-07-11

**Authors:** Daniela Djurovic Koprivica, Tatjana Puskar, Igor Budak, Mario Sokac, Milica Jeremic Knezevic, Aleksandra Maletin, Bojana Milekic, Djordje Vukelic

**Affiliations:** 1 Faculty of Medicine, University of Novi Sad, 21000 Novi Sad, Serbia; daniela.djurovic-koprivica@mf.uns.ac.rs (D.D.K.); tatjana.puskar@mf.uns.ac.rs (T.P.); milica.jeremic-knezevic@mf.uns.ac.rs (M.J.K.); aleksandra.maletin@mf.uns.ac.rs (A.M.); bojana.milekic@mf.uns.ac.rs (B.M.); 2 Faculty of Technical Sciences, University of Novi Sad, 21000 Novi Sad, Serbia; budaki@uns.ac.rs (I.B.); vukelic@uns.ac.rs (D.V.)

**Keywords:** 3D scanning, geometrical accuracy, CAD inspection, implants, polymer materials, impression materials

## Abstract

The paper presents the influence of impression methods, polymer materials, and implant angulation on the accuracy of the definitive working model for the production of implant-supported dental restorations, based on the analysis of results obtained using different impression methods, materials, and parallel and angulated implants. The study findings indicate that all aforementioned factors impact the accuracy of the definitive working model. Specifically, 20° implant angulation in relation to the vertical plane has a greater impact on the impression accuracy compared to parallel implants. The open and splint method in combination with addition silicone, as well as the splint method and polyether combination yielded more accurate results when using implants under 20° angulation compared to other method and material combinations. The splint method in combination with addition silicone resulted in the smallest mean deviations from the center of the parallel implant base compared to other combinations of methods and materials. Analysis results further revealed statistically significant differences in the measured indicators across impression methods, implants, and polymer materials.

## 1. Introduction

Owing to the technological advancements in the medical field, and the extensive research-based evidence confirming the effectiveness of implant prosthetics, this scientific and clinical discipline has gained popularity in recent decades [1]. Meta reviews of the findings yielded by the available longitudinal clinical trials on the long-term survival rate of endoosseous implants indicate that implant-supported single crowns (SCs), implant-supported fixed dental prostheses (FDPs), and implant-supported removable partial dentures (RPDs) are safe and predictable treatment methods with high success rates [2,3,4]. Although such favorable scientific evidence has influenced the wide application of implantology and implant prosthetics, a certain percentage of technical and biological complications of different character and etiology should not be neglected [2,4,5], as they indicate that a careful and thoughtful approach is needed when planning and fabricating superstructures supported by implants.

Implant-supported dental restorations that are produced by the conventional method are made indirectly, on a model that should faithfully reproduce the morphology, position, and interrelationships of the remaining teeth, implants, residual alveolar ridges, and surrounding soft tissues [1]. Clinical and laboratory procedures involved in the creation of implant prosthetics are numerous and highly demanding. Owing to this inherent complexity, each phase can lead to positional distortions and a consequent mismatch between implant-supported replacement and abutment [4,6]. It was demonstrated that a certain level of misalignment can be tolerated by the peri-implant bone, without adverse biomechanical consequences [6,7]. However, the minimum threshold of biological tolerance to the mismatch between superstructures and implant components has not yet been scientifically defined or quantified [7]. Based on the available information, and the methods presently used for assessing misalignment, the level of biological tolerance cannot be determined accurately and precisely, and all current data on the permissible deviation limits are more empirical than scientific [6,7,8]. The longevity of implant-supported restorations is closely related to the ability of the implant system to withstand occlusal loads without excessive stress or production of extensive forces in the peri-implant osseous region and the abutment-restoration junction [4]. A review of the available literature revealed that the goal of absolute passive fit is still almost impossible to achieve [6,7]. The problems arising due to the lack of completely passive restoration-abutment alignment are more pronounced in screw-retained implant-supported prostheses than in cement-retained ones, because abutment accuracy is more relevant in the former cases owing to the limited space for cement.

An ample body of research confirmed that screw-retained restorations carry a greater risk of complications, primarily of a technical nature, such as crew loosening or loss, fractures in the aesthetic part of the restoration due to reduced occlusal surface, screw location in an unfavorable location, mesostructure fractures, etc. [7,8,9]. In their study on the assessment and stratification of risk factors for peri-implant complications, which included 1275 patients, De Araujo Nobre et al., (2016) noted absence of passive fit between the implant-supported restoration and abutment and the optimal relationship between the screw components as one of the most common causes of adverse outcomes. These authors also stated that these issues can be mitigated by improving the clinical procedures adopted in the development of restorations [5].

Accurate and precise impression is the most important link in the chain of prosthetic rehabilitation of the stomatognathic system with the help of implant-supported dental restorations. This clinical procedure in the construction of superstructures on implants has a direct impact on the relationship between implant-supported restorations and abutments [10,11].

A highly pertinent topic in this context is the loading and adaptation of implants and peri-implant tissue to the stresses that arise as a result of the orofacial system functions. Many factors can affect the behavior of the implant and the receiving region, such as surgeon experience [12], and are increasingly being studied. The development of dental and implant-supported restorations using computer-aided systems has undergone exponential growth and development in the last few decades [13]. Owing to the rapid technological advances, and the emergence of new materials and processes for the production of dental restorations, the current trend in dental technology is to achieve a fully digital dental restoration production process, from the planning stage to the final restoration [14]. The process of computer-aided restoration production based on CAD/CAM technology involves data collection, data processing, and production [15]. Impressions are taken using digital intraoral cameras, which are increasingly being used in clinical practice [16,17,18,19], or through extraoral 3D scanning of the plaster model, obtained by conventional impression-taking method [20]. Moreover, the application of digital evaluation tools allows for more accurate dental implant placement [12], whereby different methods can be used to assess the 3D accuracy of dental implant positions [21]. The intraoral 3D scanning method significantly shortens the time taken to obtain impressions, and eliminates the need for conventional impression-taking methods and model casting, which should compensate for all potential errors that accompany conventional methods, thus increasing the overall accuracy [22,23]. Whatever 3D scanning method is used, the aim is to obtain a virtual 3D model and input the gathered data into an appropriate software for further processing and production. Although the future of dentistry lies in digitization and computer-aided fabrication, conventional impression-taking and fabrication methods are still widely applicable in clinical practice and need to be compared with digital methods through further studies. Due to the aforementioned factors pertaining to implant-supported restorations and the issues they cause in the creation of implant superstructures, they have been a subject of extensive research with the goal of achieving as intimate restoration-abutment alignment as feasible.

The objective of the present study is to contribute to this line of research by providing a strategy for producing a more accurate definitive working model, and thus a better fit between the prosthetic superstructures and implants. As a part of this work, a methodology that can be used for obtaining impressions of both substructures (teeth and implants) in a single phase is demonstrated by applying it to a specific clinical case, along with determining the degree of deviations that should not jeopardize the reconstruction longevity. Moreover, for both parallel and angulated implants, in addition to geometrical analysis of teeth-abutments by CAD inspection and measurement of spatial deviations of the implant base from the implant center on the nominal model (linear deviation), analysis of the geometrical deviation between the scanned implant lateral axis and the nominal master model (angular deviation) was performed.

## 2. Materials and Methods

**Figure 1:**
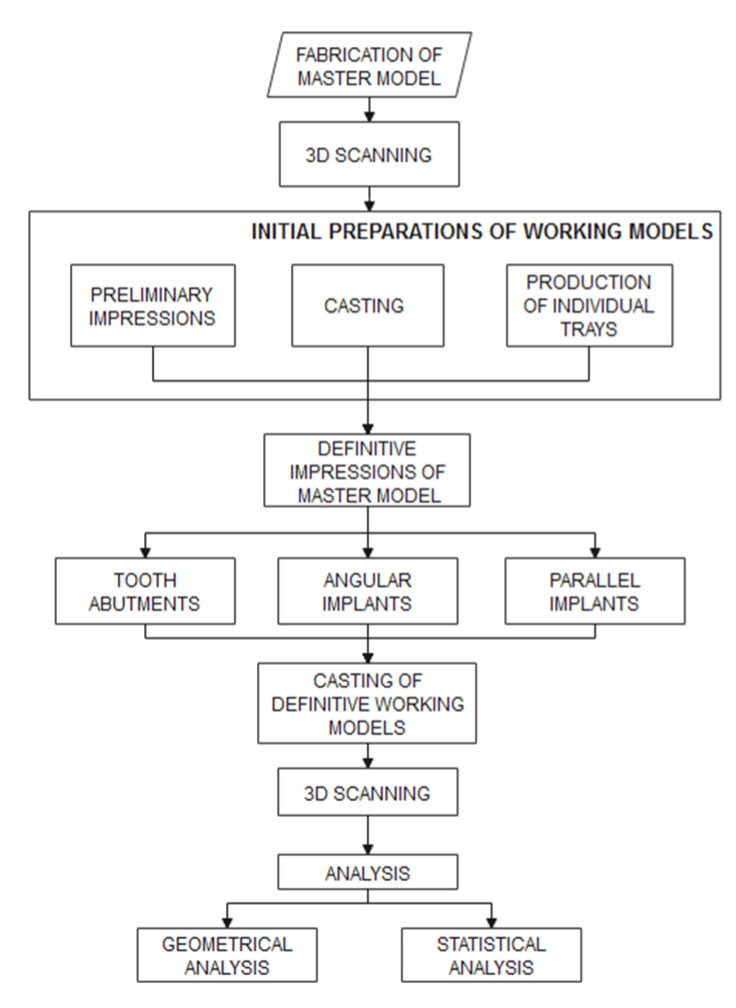
Methodology workflow.

The research reported here was performed according to the workflow shown in Figure 1. In the first step, a plaster master model was obtained, which was subsequently subjected to 3D scanning. As a part of the initial preparations, preliminary impressions of master model were obtained and cast, followed by the production of special trays. Next, definitive working model impressions were created, which were comprised of three groups, tooth abutments, parallel implants, and angulated implants. Finally, 3D scanning of all samples was performed to facilitate their dimensional and statistical analysis. All prosthodontic procedures, which included tooth preparation, preliminary and all definitive impressions were performed by dental prosthodontics specialist. The implant placement was entrusted to an oral surgery specialist, and the preparation of dental impression materials was performed by a well experienced dental nurse. Casting of working models as well as scanning of master model and replica models were performed by experienced dental technician.

### 2.1. Fabrication of Master Model

**Figure 2:**
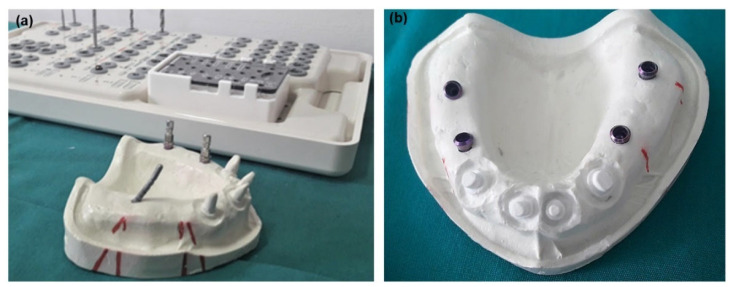
Showing (**a**) implant placement and (**b**) finished maxilla master model.

The reference master model was designed to mimic the very common clinical situation of partial edentulism (Kennedy Class I) in the upper jaw, with presented central incisors and canines (11, 13, 21, and 23), which is an indication for the placement of two implants in the posterior region of both sides of the jaw to complete the dental arch. In such clinical conditions, the regions selected for implant placement are most often the first premolar and first molar teeth regions, because often anatomical conditions, such as a low maxillary sinus floor, do not allow placement of a distal implant in the second molar region without prior surgical intervention. The remaining teeth were prepared to accept the ceramic front bridge (range 13–23), while two implants were installed in the lateral edentulous areas on each side in the region of the first premolar (tooth regions 14, 24) and the first molar (tooth regions 16, 26). On one side, the longitudinal axes of both implants were parallel and perpendicular to the horizontal plane, while on the opposite side, the anterior and the posterior implants were positioned at a 20° angle to the vertical plane (anterior distally and posterior mesially), to ensure their convergence. The model was produced by casting a factory mold of the maxilla model with full dentition in hard plaster (type 3), which was subsequently remodeled by cutting all teeth except the central incisors and canines into a model that mimics the given clinical case of partial edentulousness. For more facile processing of the remaining teeth and subsequent digitization, the adapted model was replicated in polyurethane resin (Mock-up frame, Zirkonzahn, Germany). Tooth preparation was performed using a special set (All Ceramic Preparation Kit, Shofu Inc., Kyoto, Japan) respecting all the principles of tooth preparation for ceramic crowns. At the very beginning, the teeth were prepared in a parallelometer (Paraskop^®^ M-BEGO, Germany), using a 4° carbide conical cutter in order to ensure the parallelism of the abutment teeth, and subsequently the demarcation step was finished on each abutment using a special drill from the set with a flat tip and a diameter of 1 mm and a high-speed water-cooled drill (POWERtorque LUX 646B, KaVo Dental GmbH, Ger-many), manually. Next, with the help of the so-called “Angulation key”, on the one side of the edentulous region, implants were positioned parallel to each other and perpendicular to the horizontal plane, while ensuring their convergence on the other side by situating them at a 20° angle to the vertical plane (Figure 2a,b). All four implants were soft tissue implants (STL, Standard Plus Implant Endosteal, diameter 4.1 mm, Regular Neck 10 mm SLA^®^, Straumann, Switzerland). At the end of master model fabrication the prepared front teeth (teeth 11, 13, 21 and 23), since the material from which the master model was made is brittle, the abutments were shortened to a level of 4–5 mm above the demarcation step to prevent breakage during many impressions, and in order to obtain a good impression of the abutment around the demarcation zone and just above which required exceptional accuracy for fitting the future ceramic restoration. That area was subsequently subjected to CAD inspection for each abutment individually.

### 2.2. 3D Digitization

**Figure 3:**
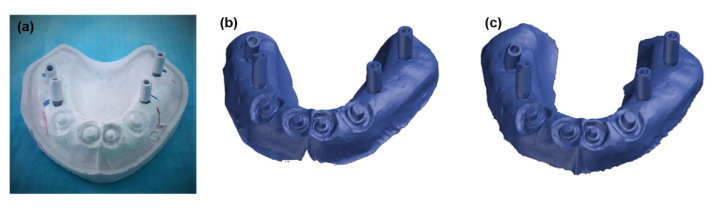
Showing (**a**) master model with screw-secured scan bodies, and the corresponding 3D reference models reconstructed by (**b**) Identica Blue and (**c**) D900L 3D scanners.

Once the master model was complete, it was subjected to 3D digitization to obtain two reference master 3D models. For this purpose, two commercial dental 3D scanners (D900L from 3Shape and Identica Blue from Medit) were used. These laboratory scanners were used because they have very similar scanning characteristics, primarily in scanning accuracy (less than 10 µm). Master model scanning resulted in two virtual master models that were later compared with the scanned replica models. For gathering data on the spatial orientation of the implants and subsequent deviation analysis, scanning abutments (Scan bodies) specifically designed for this purpose were employed (Figure 3a). Figure 3b,c show 3D models produced by these commercial 3D scanners.

### 2.3. Preliminary Preparations

**Figure 4:**
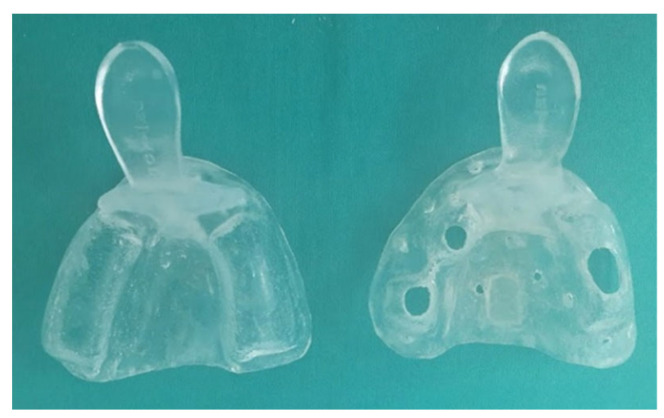
Showing closed (**left**) and open (**right**) tray for obtaining definitive impressions.

In this phase, impressions of the master models were obtained using standard perforated tray and irreversible hydrocolloid (Hydrogum alginate, Zhermack, Italy) to allow the preliminary working models to be cast. Based on the impressions taken with the irreversible hydrocolloid, the two preliminary models in hard plaster were cast-type 3 (Quickstone Laboratory Stone-Whip-Mix Corporation, USA). On these preliminary models, individual trays (3 closed and 6 open) were made using thermoplastic acrylic foil (Biocryl C, Clear, 3 mm, Scheu-dental, Germany), for the purposes of producing definitive impressions, which are shown in Figure 4.

### 2.4. Definitive Impressions

For definitive impressions, impression accuracy was assessed using three main control groups—tooth abutments on the master model, angulated implants on the master model, and parallel implants on the master model—each comprising samples obtained by 3D digitization using two 3D scanners.

Two types of polymer impression-taking materials were used, addition silicone/polyvinyl siloxane-PVS (Elite HD + Light Body Fast Set, Zhermack, Italy) and polyether-PE (Soft Monophase Impregum Penta, 3M ESPE, USA). In total, 30 master model impressions were produced (10 for each of the aforementioned methods), and all impressions were taken at the implant level. Depending on the technique performed, the appropriate manufacturer-supplied tools were used. Table 1 provides the details of the experimental groups and the experimental plan.

**Figure 5:**
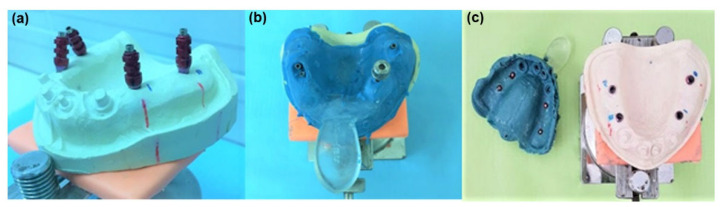
Direct (open) impression method: (**a**) twisted transfers, (**b**) addition silicone impression in the screw unfastening phase, and (**c**) polyether impression.

For the direct methods with and without transfer splinting, transfers corresponding to the implant types from the original manufacturers’ sets were used (i.e., Straumann^®^, Tissue level, Regular neck, synOcta open-tray impression cap, with integral guide screw, red), as shown in Figure 5a–c.

**Figure 6:**
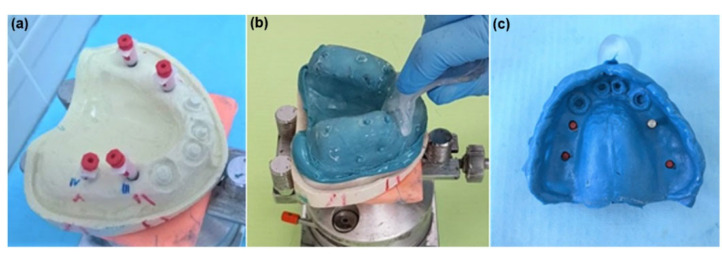
Indirect (closed) click impression method: (**a**) mounted transfers, (**b**) polyether impressions, and (**c**) addition silicone impression after separation.

The indirect method involved the use of a transfer system from the original set of manufacturers that uses snap-on plastic transfer caps designed to remain in the impression after binding of the material (Straumann^®^, Regular neck, synOcta closed-tray impression cap and positioning cylinder, red) (Figure 6a–c).

Impressions were performed by the same therapist throughout the whole experiment, during which the pressure and removal force were approximated by the therapist’s tactile sensitivity method, in order to mimic the clinical conditions of taking impressions in implant prosthetics as closely as possible.

### 2.5. Definitive Working Model Casting

**Figure 7:**
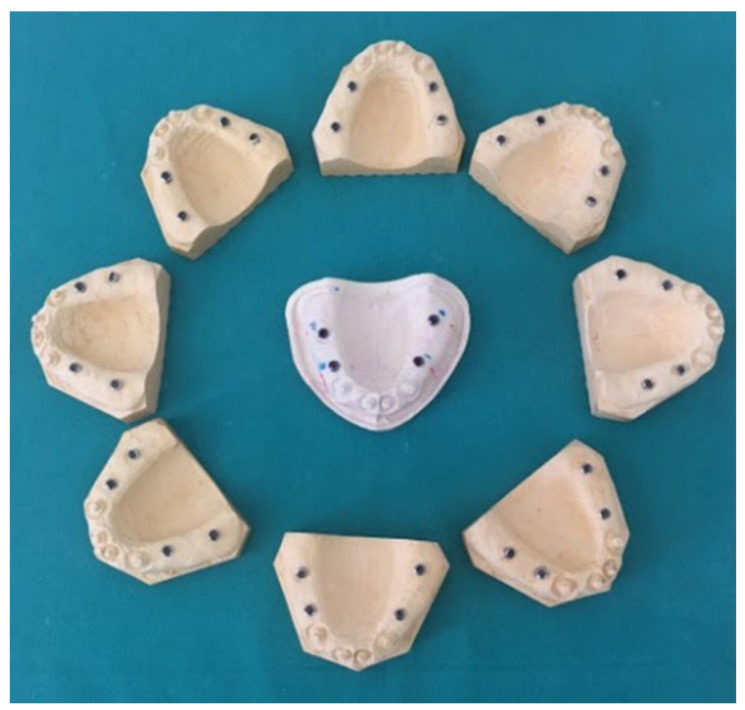
Definitive working models with embedded implant body analogs.

Once all impressions were made, each of them was cast in superhard plaster, type 4 (GC Fujirock^®^, Japan). Thirty definitive working models with embedded implant body analogs were obtained for experimental 3D digitization and further deviation analysis (Figure 7).

### 2.6. 3D Digitization of Definitive Working Models

**Figure 8:**
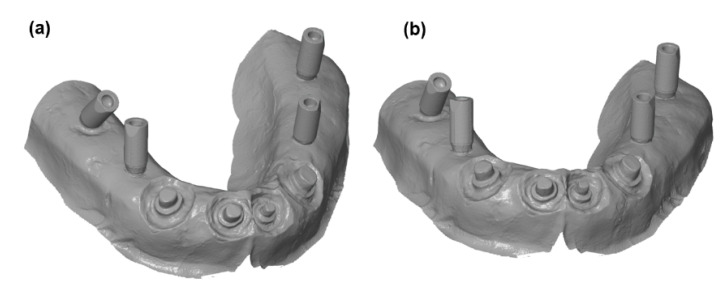
Digitized 3D models obtained using (**a**) Identica Blue and (**b**) D900L commercial 3D scanners.

To perform geometrical analysis, 30 experimental replicas of definitive working models were scanned using two commercial 3D scanners. To allow the deviations in the spatial orientation of the implants on the experimental working models to be analyzed, a “scan body” was fixed on each analog of the implant body prior to 3D scanning. Three-dimensional digitization of definitive working models was performed using the same procedure and the same parameters as in the case of master 3D model scanning. The resulting 3D models of definitive working replicas were obtained using Identica Blue and D900L commercial 3D scanners, as shown in Figure 8.

### 2.7. Geometrical Analysis

**Figure 9:**
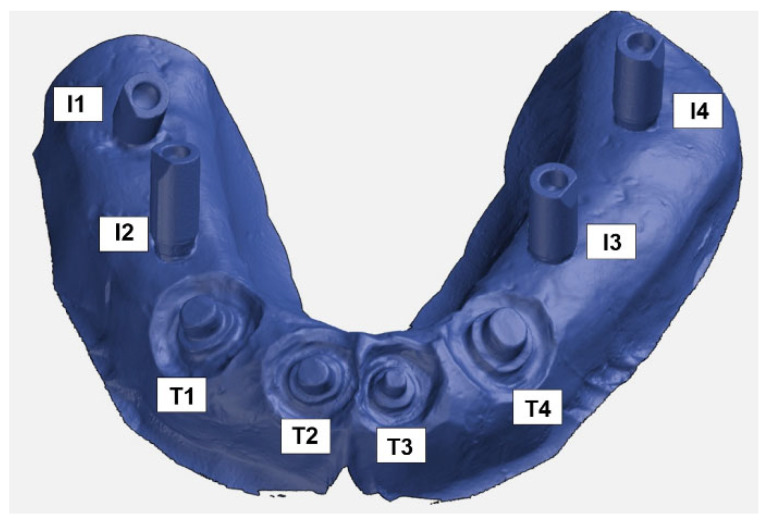
Parameters included in the analysis: T1–T4 (teeth) and I1–I4 (implants).

Prior to the analysis of the geometrical deviations, a CAD inspection of digitized working models was conducted. The software used for this analysis was GOM Inspect v2021. This analysis was performed on the tooth abutments and implants separately. To clearly present the results, the parameters included in the analysis were defined and clearly marked on the reference (master) and experimental 3D models. The tooth abutments were denoted with the symbol T1, T2, T3, and T4, while “I” was chosen for the implant abutments, whereby angular implants were labeled I1 and I2, and parallel implants I3 and I4 (Figure 9).

**Figure 10:**
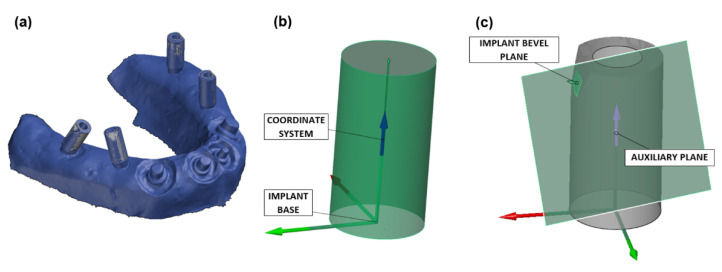
Showing (**a**) implant positioning on the master model using best-fit method, (**b**) orientation of the local coordinate system of the master 3D model, and (**c**) determination of the Y axis direc-tion in the local coordinate system.

In the geometrical analysis of implants, the master model was used to analyze the deviations in scanned experimental 3D models obtained by different impression methods. Since the master model itself was obtained by 3D scanning, the base positions of the angulated and parallel implants could not be determined definitively. To ascertain the exact position of the implant base and enable accurate comparison and analysis of deviations on the experimental 3D models, implant 3D model was imported from the Straumann library and was superimposed on the master model using the best-fit method. This method consists of finding common points between the master 3D model and implant 3D model and aligning their geometry using iterative closest point (ICP) algorithm. The import and positioning procedure was repeated for all implants (Figure 10a). A local coordinate system was created on each imported implant and was placed in the centers of the circles that form the implant base (Figure 10b). To fully determine the direction of the Y axis, an auxiliary plane containing the implant axis that is perpendicular to the implant bevel plane was created (Figure 10c).

**Figure 11:**
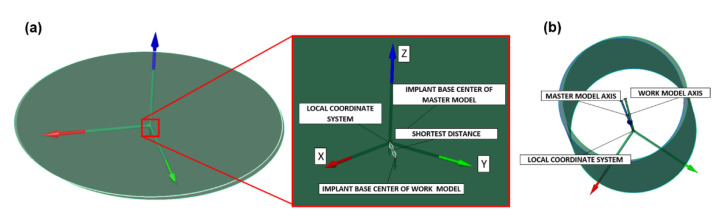
Showing (**a**) measurement of the deviation of the base of the scanned implant and the master model (linear deviation) and (**b**) analysis of the deviation of the scanned implant lateral axis relative to the lateral implant axis (angular deviation) of the master model implant.

By establishing the local coordinate system, the deviation of the center of the experimental 3D model base could be examined in relation to the origin of the corresponding local coordinate system of the master model. The analysis included deviations in the directions of all three axes (x, y, z), as well as the shortest distance between the base center and the local coordinate origin (x, y, z), as shown in Figure 11a. The same procedure was performed for all 3D models generated by both 3D scanners. The geometrical deviation analysis of the lateral axis of the scanned implant in relation to the lateral axis of the master model implant was performed by calculating the absolute angle between the axes (Figure 11b).

**Figure 12:**
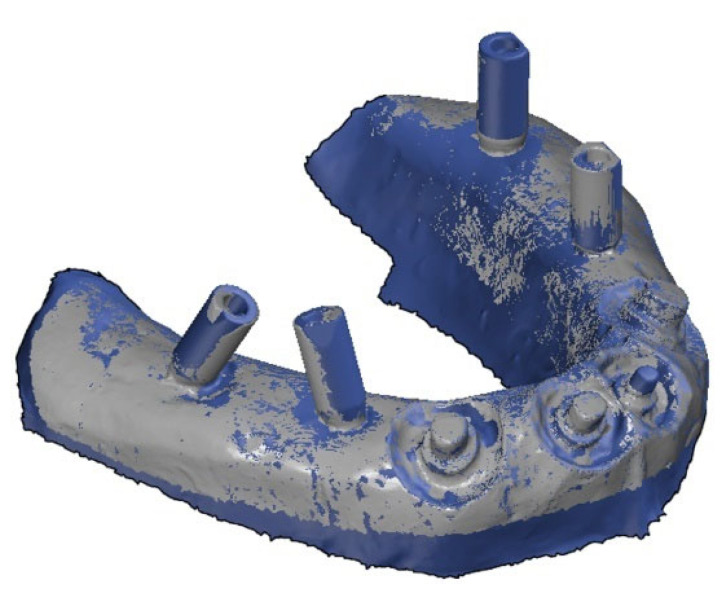
Alignment of the experimental (gray color) and master (blue color) 3D models using best-fit method.

When analyzing the experimental model deviations, superimposition was also necessary to ensure their correct alignment with the virtual master model. The best-fit method was also used for this purpose (Figure 12). The same procedure was performed for each individual experimental and master 3D model.

### 2.8. Statistical Analysis

Based on all data yielded by the dimensional analysis, a database was created, which was processed for the purpose of statistical analysis by applying the SPSS 20.0 commercial software. In this work, the aim was to establish existence of any statistically significant differences among the relevant misalignment indicators depending on the impression methods, implants, and materials used. In all analyses, *p* < 0.05 was taken as the threshold of statistical significance. Determination of statistically significant differences of deviations from the center and the angle of implant deviations was analyzed using Multivariate Analysis of Variance (MANOVA) and Analysis of Variance (ANOVA) for all combinations of methods, implants, and materials.

## 3. Results

### 3.1. CAD Inspection and Implant Base and Lateral Axis Deviation Measurements

**Figure 13:**
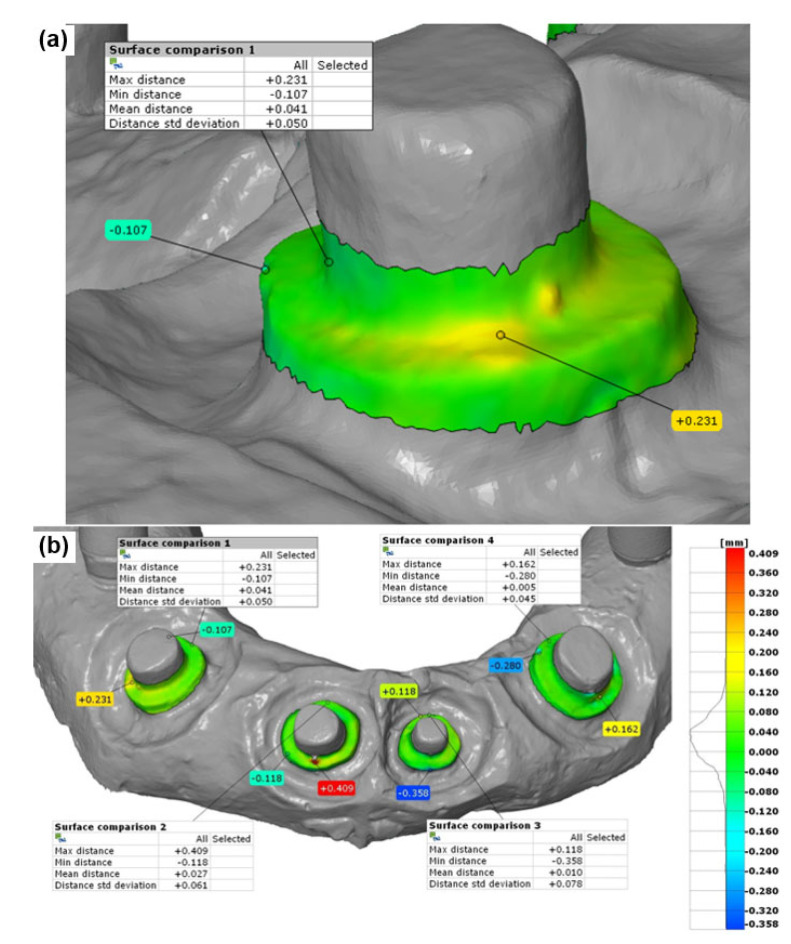
Showing (**a**) CAD inspection analysis of the selected area on one tooth and (**b**) analysis of all teeth (T1–T4).

In the CAD inspection of the tooth abutments, the zone of step transition (demarcation zone) was analyzed for each abutment individually (Figure 13a,b). The geometrical deviation analysis included assessment of the deviations of the surfaces defined on the experimental 3D model from the corresponding surfaces of the reference 3D model (based on the distance of the vertex of the triangle in STL data format). The CAD inspection results that were taken into account in further analyses included maximum deviation, minimum deviation, mean deviation value (for the defined abutment demarcation zone), and standard deviation value. This CAD inspection procedure was performed for all samples obtained by both 3D scanners.

As this analysis resulted in an extensive dataset, Table 2 only shows the CAD inspection results related to the PVS material (sample no. 1) for all four teeth (T1–T4), along with the analyzed parameters, namely maximum deviation, minimum deviation, mean deviation (for the defined teeth demarcation zone), and standard deviation value.

Taking into account the method adopted for measuring the deviation of the implant base and the lateral axis of the scanned implant, shown in Figure 10 and Figure 11, respectively, a 3D model analysis was performed for all samples scanned by both 3D scanners, where data were collected for both angulated (I1 and I2) and parallel (I3 and I4) implants.

Table 3 shows the deviation analysis results for the open impression method with PVS material obtained using Identica Blue 3D scanner (sample no. 1) for all four (A1–A4) implants, along with the analysis of the distance of the scanned implant abutment base relative to the nominal implant abutment base and analysis of lateral axis angles relative to the base.

### 3.2. Implant Spatial Deviation Results

**Figure 14:**
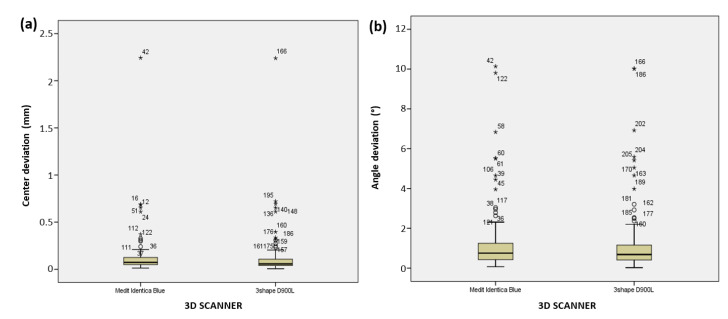
Graphic display of the analysis results depicting the deviation from (**a**) the center and (**b**) the angle of implant deviation for both 3D scanners.

Due to the large volume of acquired data, and with the aim of grouping the results, the analysis initially focused on the differences arising between the use of two 3D scanners. Figure 14a,b shows the results related to the linear and angular deviations for both 3D scanners. Here it can be seen how implant deviations are expressed when two 3D scanners are used. It shows the distribution of deviations for measurements of the implant’s center and angle obtained from two 3D scanners.

As several results (from Figure 14) had outliers, the data were modified. The most extreme cases, which likely arose due to random measurement errors during 3D scanning, were excluded from further analysis. The excluded data (pertaining to the same measurement) were replaced by the next maximum value from the remaining results of that group. In total, six data points (three from each scanner) required removal from further analysis: two of which were related to the open method and four to the closed method, two were from the group of parallel implants and four from the group of angular implants, and two were from the addition silicone group and four from the polyether group.

After the preliminary analysis, 3D scanner accuracy was subjected to the multivariate analysis of variance. The results revealed absence of statistically significant differences in the linear and angular deviations between the two scanners F(2,231) = 0.572; *p* = 0.565. This finding statistically justified the exclusion of 3D scanner as a grouping variable, allowing the aggregation of data from both scanners prior to further analysis. The groups were thus divided into subgroups, based on the comparisons performed as a part of this research:

- Three groups based on the applied impression method (open, closed, and splint),
- Two groups based on implant type (angulated and parallel),
- Two groups based on the impression material (additive silicone/PVS and polyether/PE).

**Figure 15:**
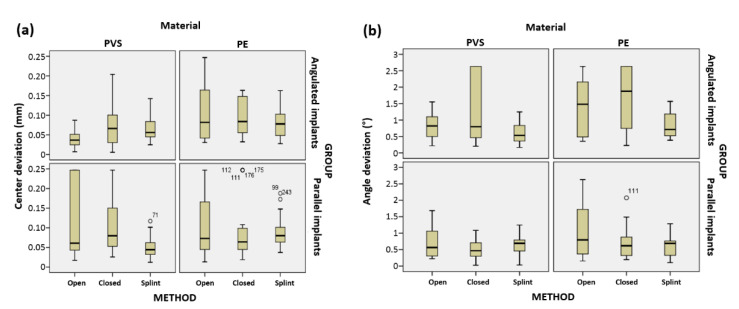
Graphical representation of the distribution of (**a**) the distance from the implant base center and (**b**) the angle of deviation.

A graphical representation of the distribution of values obtained for the linear and angular deviation, according to the previously defined groups, is shown in Figure 15a,b.

**Figure 16:**
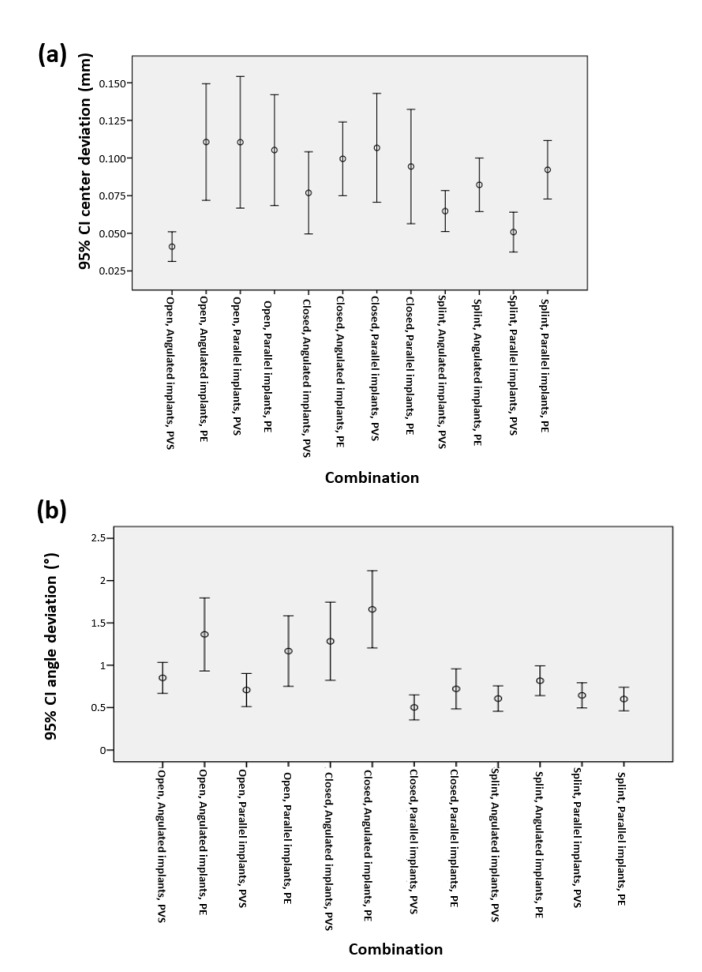
Graphical representation of (**a**) the mean deviation from the implant center (mm) (**b**) and the mean deviation angle (°) for all possible combinations of impression method, implant group and material type.

As evident from Figure 15, no extreme results were noted for any combination of groups or for any observed value. Figure 16a,b shows the graphical representations of the mean deviation from the implant center and the mean deviation angle (°) for all possible combinations of impression method, implant group, and material type.

It is evident from Figure 16a that for the angulated implant subgroup, the mean linear deviations in the Open/PVS combination were statistically significantly smaller compared to Splint/PE, Closed/PE and Open/PE, but were comparable to the values obtained for the Splint/PVS and Closed/PVS combinations.

Based on Figure 16b, in the angulated implant subgroup, the Splint/PVS, Splint/PE, and Open/PVS combinations had a statistically significantly lower mean angular deviation than the Closed/PVS, Open/PE, and Closed/PE combinations, for which the values were comparable. In the parallel implant subgroup, the Closed/PVS, Splint/PE, Splint/PVS, Open/PVS and Closed/PE combinations did not exhibit statistically significant differences in the mean angular deviation, but the values obtained for the Open/PE combination were statistically significantly higher than those related to all other combinations of methods and materials.

### 3.3. Tooth Abutments Deviation Results

**Figure 17:**
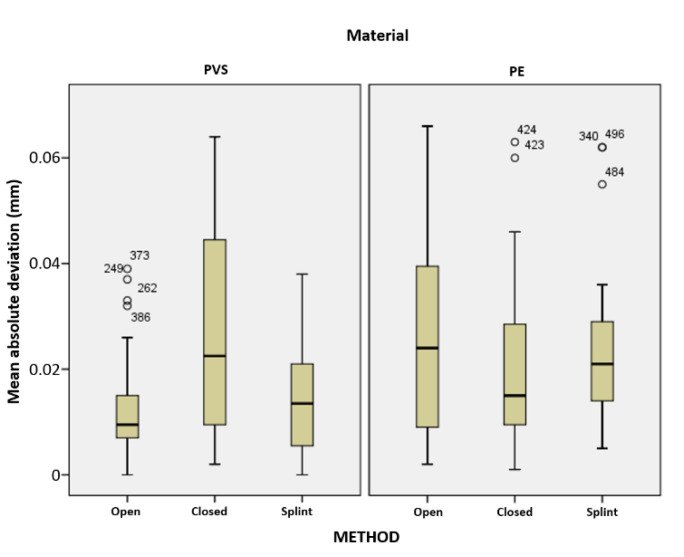
Graphical representation of the distribution of mean absolute deviations for the tooth abutments according to the method and material used.

The preliminary data processing of the results related to the deviation of the scanned tooth abutment surfaces also included a comparative analysis of the measurements obtained by both 3D scanners, in order to enable the aggregation of data related to all analyzed samples. The values included in the analysis of scanned abutments were the maximum and minimum deviation, i.e., their mean value (mean absolute deviation). Figure 17 shows a graphical representation of the distribution of absolute mean deviations for the tooth abutments according to the method and material used.

**Figure 18:**
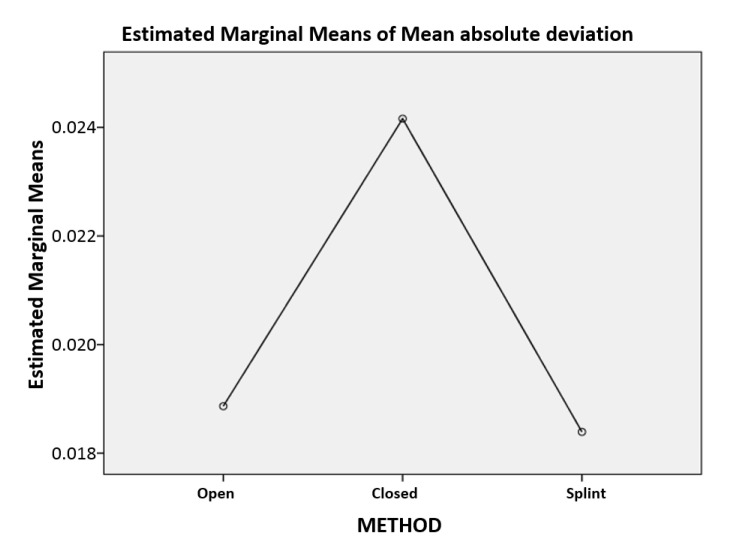
Mean absolute deviation (mm) according to the impression method.

Analysis of variance results indicated the presence of statistically significant differences in the mean absolute deviation according to both impression method and material, with F(2,213) = 3.452, *p* = 0.033, and F(1,213) = 7.271, *p* = 0.008, respectively. The interaction between the method and the material also proved to be statistically significant, with F(2,213) = 9.739, *p* < 0.001. When a pairwise comparison was performed, statistically significant differences between closed and open methods (*p* = 0.012) and closed and splint methods (*p* = 0.007) also emerged. Specifically, the mean absolute deviation related to the closed method was statistically significantly larger than that obtained for the other two methods (Figure 18).

## 4. Discussion

As the acceptable misalignment between the finished implant-supported restoration and the abutment has not been scientifically quantified, no limit for the detected deviations was set in the present study, and all measurements were included in the statistical analysis. Namely, the boundaries set by various researchers for the measured deviation values, considering the values below the set limit to be biologically tolerable, range from 10 μm to 150 μm, and were chosen arbitrarily, guided by the empirical evidence regarding the degree of inaccuracy that would not cause complications [7,24]. The innovativeness of the methodology presented in this work relative to those reported in extant literature stems from the fact that for the first time, the accuracy of tooth abutment and implant impressions was simultaneously examined during the complete reconstruction of the dentition with combined teeth and implant-supported fixed prosthetic restorations, which was also accompanied by preliminary research on this topic [25]. In this work, the deviations in implants and tooth abutments were analyzed and interpreted separately, due to the adoption of different geometrical analysis methodologies for each parameter. Comparative analysis of two commercial 3D scanners (Identica Blue and D900L) revealed that both yielded comparable results, allowing the datasets produced to be merged in further analyses. When processing the implant misalignment results, the data obtained for the deviation from the center (linear deviation) and the deviation angle (angular deviation) were analyzed. The findings yielded by the analyses conducted on parallel and angulated implants will contribute to the easier detection and elimination any impression-related errors, and would thus facilitate attainment of a more accurate prosthetic restoration fit. Furthermore, by evaluating the accuracy of conventional and modified impression methods, this work will aid in a more effective establishment of clinical indications for their use in practice. Finally, through the examination of a large number of parameters, along with the use of three impression methods, two impression materials, and three types of supporting sub-elements (teeth, parallel implants, and angulated implants), several significant results in the field of implant prosthetics was obtained.

Analysis of the three impression methods revealed that the application of the splint impression method resulted in the smallest mean deviations from the center of the implant base compared to the open method without transfer splinting and the closed click method, which yielded comparable results. Moreover, findings yielded by analyzing the mean angle of deviation indicated that the splint method was most accurate, while the accuracy of the remaining two methods was comparable. The same conclusions were reported by Stimmelmayr et al., for the placement of four implants in edentulous jaws in several studies [26,27,28]. The results obtained in the present study also concur with those reported by Vigolo et al., who, based on three investigations, concluded that transfer splinting exerts a positive effect on the impression accuracy [29,30]. In their work, Papaspyridakos and colleagues compared implant-level impressions, and demonstrated that the splint method was more accurate than the non-splint alternative [31,32,33,34]. The absence of statistically significant differences between non-splint open method and closed click method, in terms of mean linear and angular deviations, coincides with the results obtained in several extant studies [35,36,37]. Nakhaei et al., who compared open and closed impression methods while using the same implant type as that adopted in the present study, also showed that the two yielded comparable results [38].

However, it is impossible to directly compare the findings obtained in the aforementioned investigations due to the differences in the implant type, number and angulation, as well as the research methodology, the impression surface accuracy assessment method, etc. The present study is original because of the presence of tooth abutments in the frontal part of the arch, as well as the extreme angulation and mutual convergence of the implants on one side.

Moreover, a significant difference in the results obtained for the implant groups was also noted, and impressions involving parallel implants were more accurate, confirming the necessity of surgical guides during implantation, in order to achieve a better-fitting restoration and improve its durability. When analyzing the data obtained by measuring the linear deviation in this comparison group, there was no statistically significant difference between angulated and parallel implants.

The analyses further revealed that implant angulation, which was adopted in the present study to facilitate comparison with parallel implants, affects the positioning of supragingival part of the implant on the replica model, which in the further course of restoration can lead to greater discrepancies between the restoration and abutment. This finding supports the evidence provided by many researchers indicating that the use of non-parallel implants adversely affects the impression accuracy [8,33,39,40]. In their 2015 study, Tsagkalidis et al., examined the limit of implant angulation above which the influence of this parameter on the impression accuracy becomes apparent, and concluded that an implant angulation of 25°, unlike parallel and implants angulated at 15°, compromises the impression accuracy [8], as confirmed in the present study. The authors of previous studies mostly concur on the degree of angulation that may undermine the impression accuracy, indicating that it should not exceed 15° [11,39,41]. Moreover, researchers claiming that angulated implants do not cause inaccuracies in impressions mostly examined implant impressions with angulation below 15° [40,42]. However, some counter-evidence indicating that even 30° angulation does not adversely impact the accuracy of the definitive impression procedure has been presented [38,43].

By comparing the implant impressions obtained using addition silicone (PVS) and polyether (PE), PVS was found to result in a statistically significantly lower mean linear as well as angular deviation. These observations are in line with the results reported by Sorrentino (2010), Kurtulmus-Yilmaz (2014), and Vojdani (2015) [11,44,45]. These findings were obtained by processing the mean total discrepancies for both analyzed parameters pertaining to implant misalignment, analyzing each group individually and conducting comparative analysis within one group. Since challenging clinical cases from the perspective of determining the most optimal impression method and material are frequently encountered in implant prosthetics, in order to expand the practical value of the present study, further pairwise analyses were conducted on all groups.

As the two main groups comprised of three subgroups (based on the impression method and material used) and one had two subgroups (the implant group), the total number of all possible combinations was 12. Thus, individual analyses were also performed for each of the measured parameters, for all possible group combinations. The findings indicated that in the angulated implant group, for the mean linear deviation, the combinations involving the open method, splint, and closed method with addition silicone yielded statistically significantly smaller deviations compared to other combinations, while there was no statistically significant difference among these three combinations.

This result also explains the absence of statistically significant differences between angulated and parallel implants in terms of the deviation from the center of the implant axis. The mean angular deviation values for the angulated implant group differed in relation to the linear deviation. When this parameter was analyzed for this group of implants, the splint and open method in combination with addition silicone, and the splint method in combination with polyether yielded the most accurate results, without statistically significant differences between them.

A conclusion that can be reached with certainty from these findings is that PVS application resulted in the smallest deviations in both parameters in the angulated implant group. These results indicate that in the clinical case presented in this work, either open or splint impression method using addition silicone would be appropriate, because this material was shown to be most reliable.

These assertions are supported by the findings reported by Sorrentino and colleagues [45] indicating that the open method in combination with addition silicone yields the most accurate impressions of angulated implants. Similarly, several authors demonstrated that the combination of splint method and addition silicone should be adopted for cases requiring 20° implant angulation [8,11]. It is also worth noting that the splint method/PE combination, in addition to being among the most accurate of methods with respect to the angular deviation, also resulted in a relatively small linear deviation. This result can be a consequence of both transfer splinting and the impression material rigidity, which restricted the range of transfer system movement during the impression-taking process. Therefore, this particular method/material combination may be suitable for angulated implants, but since significantly better results were obtained using PVS relative to PE in terms of the total deviation in both parameters, it is not superior to the previously discussed combinations.

The closed method/addition silicone combination, which was characterized by similar accuracy in terms of linear deviation, cannot be recommended with certainty for implants of this angulation, as it produced significantly larger discrepancies in the mean angular deviation. This issue was likely caused by the movement in the transporters themselves when separating the impression from the imprinted surfaces, which resulted in greater discrepancies in the coronary parts than in the gingival parts that rest on the implant neck. As the splint method produced the smallest overall deviations in both measured parameters, and stood out as one of the most accurate in the group of angulated implants, which is also true for PVS material; it can be concluded that this combination of method and material is the right choice for implants with 20° angulation with respect to the vertical plane. In the group of parallel implants, the splint method in combination with PVS material resulted in statistically significantly smaller discrepancies in the average deviation from the implant axis center compared to all other combinations, which again points to the previous conclusion that the splint method/PVS material is the most accurate combination for both parallel and non-parallel implants [11]. In contrast, when the method/material combinations were compared with respect to the average deviation angle for the group of parallel implants, none of the combinations yielded statistically significantly better results than the others, but the open method combined with PE produced the greatest discrepancies. The extreme mean angular deviation obtained for the open/PE/parallel implant combination may potentially be attributed to the difficulties in untwisting the transfer system during the impression-taking process, or twisting the analog once the impression was obtained, which probably led to undesirable micro movements of the transfer system within the impression. It is assumed that such issues were prevented by combining the splint method with both impression materials, as the solid acrylic block used in this case prevented carrier movements.

Thus, considering the values of both observed parameters for the parallel implant group, it can be concluded that the splint method in combination with PVS is a good choice for transferring the position of the implant body to the definitive working model, and that the combination of method and material does not affect the average deviation angle, with the exception of the aforementioned combination which produced the largest discrepancies. Although the findings obtained for the open/PE combination might be due to the reasons suggested above, they could have arisen as a result of errors during the printing phase or digitization process, which precludes any definitive conclusions about the complete inferiority of this combination relative to other combinations of methods and materials examined here.

Most studies on the implant prosthetics impression accuracy are performed in vitro, which carries the risk of the results obtained being influenced by a wide range of parameters related to the experiment that may be difficult to control. In addition, as there is no widely accepted protocol or methodology to guide the researchers, reaching consensus on the accuracy of methods and materials for a particular implant type, number, and angulation based on the obtained findings is extremely challenging. Therefore, in vitro methodologies for assessing the impression accuracy have numerous advantages, because all aggravating factors that exist in the oral cavity and can affect the impression accuracy can be avoided. The extenuating conditions, such as humid environment and difficult manipulation in the mouth, in addition to the above factors that make a clinical research method on this topic difficult to implement, may increase the likelihood of errors in in vivo impression-making process, thus reducing the accuracy of the final model compared to that obtained under in vitro conditions.

This justifies the decision made in this study to include in the analysis deviation parameters pertaining to the real surfaces of the master model, as in vitro measurements are, on average, expected to produce smaller misalignments than those attainable under clinical conditions. The conclusions drawn from all possible combinations of the main groups compared in this study provide additional information on the impact of the applied method and impression material for parallel and non-parallel implants on the accuracy of the definitive working model used in the design of implant-supported restorations. This, in turn, may facilitate the selection of an adequate method for producing implants that for whatever reason, need to converge with each other under extreme angulations, as well as for ideally parallel implants.

As a part of this study, we also examined the joint influence of the impression method and material on the accuracy of the imprinted surfaces of tooth abutments in the frontal part of edentulous maxilla during simultaneous impression for a given clinical case. The same analytical approach as for the implants was adopted to assess the resulting deviations. The preliminary data processing also confirmed that the two scanners employed in this work yielded consistent results, allowing the information obtained in both cases to be merged into a single dataset. Therefore, in the subsequent analysis, mean absolute deviation in the demarcation zone in relation to the nominal geometry was adopted. Overall, our findings indicate that splint and open method yielded comparable results, while producing statistically significantly smaller mean absolute deviations compared to the closed impression method. With respect to the same parameter, PVS was superior to PE.

Consequently, it may be advisable to use addition silicones rather than other polymer impression materials in the reproduction of surfaces captured by impressions, as suggested by other authors [46,47]. However, direct comparisons of these two material types when creating impressions of tooth abutments have rarely been conducted, and the few available cases pertain to implant prosthetics impressions. Accordingly, further research is required in order to establish precise criteria for the impression material selection in conventional fixed prosthetics as well as implant prosthetics. Nonetheless, in this study, tooth abutment impressions based on PVS were more accurate than those based on PE, and this observation coincides with the conclusion reached in relation to impressions for both parallel and angulated implants.

## 5. Conclusions

Based on the results obtained in this work, it can be concluded that the impression method, implant angulation, and the impression material type affect the accuracy of the definitive working model used for the production of implant-supported dental restorations. On basis of the research presented, it can be concluded that the split method, in combination with addition silicone, could be the combination of choice for the clinical case, because in total it had the smallest deviations and stood out as the most accurate. Additionally, the closed impression technique was the most inaccurate, especially in combination with polyether materials and angulated group of implants. With all this in mind, one of the directions of future research will be to deal in depth with polymer characteristics and their influence on clinical outcome.

Moreover, owing to the rapid technological advances in the production of prosthetic restorations, which involves the application of a completely digital production flow, future research will also include a comparison of the accuracy of intraoral digital impressions and the latest modified conventional impression techniques, all with the aim of obtaining the most accurate definitive working model and thus a more accurate fit of the prosthetic superstructure.

## Figures and Tables

**Table 1:** The experimental group characteristics and the experimental plan for the three main control groups.

	Tooth Abutments	Angulated Implants	Parallel Implants
Direct open-tray impressions without transfer splinting using addition silicone	X	X	X
Direct open-tray impressions without transfer splinting using polyether	X	X	X
Indirect closed-tray impressions using the click method and addition silicone	X	X	X
Indirect closed-tray impressions using the click method and polyether	X	X	X
Direct open-tray impressions using the splint method and addition silicone	X	X	X
Direct open-tray impressions using the splint method and polyether	X	X	X

**Table 2:** CAD inspection results for all four teeth (T1–T4) based on open impression method using PVS material (sample no. 1) and both 3D scanners.

Sample No.	Name	Group	Min. Distance [mm]	Max. Distance [mm]	Standard Deviation [mm]	Mean Value [mm]
1.	Open (PVS) Identica Blue	T1	−0.183	+0.498	+0.087	+0.037
		T2	−0.213	+0.176	+0.047	+0.015
		T3	−0.222	+0.202	+0.028	+0.013
		T4	−0.324	+0.354	+0.057	+0.008
**Sample No.**	**Name**	**Group**	**Min. Distance [mm]**	**Max. Distance [mm]**	**Standard Deviation [mm]**	**Mean Value [mm]**
1.	Open (PVS) D900L	T1	−0.261	+0.546	+0.093	+0.039
		T2	−0.187	+0.521	+0.068	+0.021
		T3	−0.080	+0.412	+0.051	+0.020
		T4	−0.326	+0.267	+0.050	+0.010

**Table 3:** Deviation analysis results for the open impression method with PVS material (sample no. 1) for angulated implants (I1 and I2) and parallel implants (I3 and I4) using both 3D scanners.

Sample No.	Name	Group	Distance from the Scanned Abutment Based to the Nominal Abutment Base [mm]				Cylinder Axis Angles Relative to the Base [°]	
			**x**	**y**	**z**	**xyz**	**xyz**	
1.	Open (PVS) Identica Blue	I1	−0.0015	−0.0212	−0.0050	0.0219	1.5520°	1°33′7″
		I2	−0.0210	−0.0421	0.0010	0.0471	0.2817°	0°16′54″
		I3	−0.0524	0.0210	0.0058	0.0568	1.1729°	1°10′22″
		I4	0.0294	0.0392	−0.0030	0.0491	0.2962°	0°17′46″
**Sample No.**	**Name**	**Group**	**Distance from the Scanned Abutment Based to the Nominal Abutment Base [mm]**				**Cylinder Axis Angles Relative to the Base [°]**	
			**x**	**y**	**z**	**xyz**	**xyz**	
1.	Open (PVS) D900L	I1	0.0028	−0.0061	0.0010	0.0067	1.4504°	1°27′1″
		I2	−0.0232	−0.0386	−0.0119	0.0466	0.2171°	0°13′2″
		I3	0.0353	0.0280	−0.0155	0.0477	0.9010°	0°54′4″
		I4	0.0178	0.0273	−0.0073	0.0334	0.3535°	0°21′13″

## Data Availability

Not applicable.
